# An HPLC-based assay for soil enzyme activities using 4-nitrophenyl substrates

**DOI:** 10.1016/j.mex.2026.104026

**Published:** 2026-07-02

**Authors:** Williams C. Iwebema, Joerg Geistlinger, Anita Kirmer, Wilfried Rozhon

**Affiliations:** a Analytical and Bioanalytical Sciences, Department of Agriculture, Ecotrophology, and Landscape Development, Anhalt University of Applied Sciences, Bernburg, Saxony-Anhalt, 06406, Germany; b Vegetation Science, Department of Agriculture, Ecotrophology, and Landscape Development, Anhalt University of Applied Sciences, Bernburg, Saxony-Anhalt, 06406, Germany

**Keywords:** Enzyme assays, HPLC, Extracellular enzymes, Organic soils, Organic matter interference, 4-nitrophenyl substrates

## Abstract

Soil enzyme activity assays are useful for assessing the biogeochemical potential of soils. Frequently, 4-nitrophenyl-labelled chromogenic substrates are used that yield yellow 4-nitrophenol (4-NP), which can be quantified by spectrophotometry at 410 nm. However, this approach is often impeded by the presence of organic matter, which causes yellow or even brownish colouring of the soil extracts, interfering with spectrophotometric measurements. In this study, we show that high-performance liquid chromatography (HPLC) is an efficient, rapid and selective method for quantifying the liberated 4-NP. The soil samples were mixed with the substrates dissolved in buffers and incubated for a suitable time period. Subsequently, the reactions were analysed by HPLC using a LiChroCART RP18 3 µm 55 × 2 mm with an eluent consisting of 20 mmol L^-1^ acetic acid in acetonitrile/water = 30/70 (v/v) at a flow rate of 0.4 mL min^-1^, a run time of 2.7 min and absorbance detection at 320 nm. This method is particularly suitable for carbon-rich soils, omitting the need for substrate-free controls, and offers excellent reproducibility while maintaining the simplicity and accuracy of traditional soil enzyme assays.

• Simple, selective and highly reproducible method for measurement of soil enzyme activities.
• Application of 4-nitrophenyl substrates.
• Efficient separation of 4-nitrophenol from humic acids.

## Graphical abstract

**Figure:**
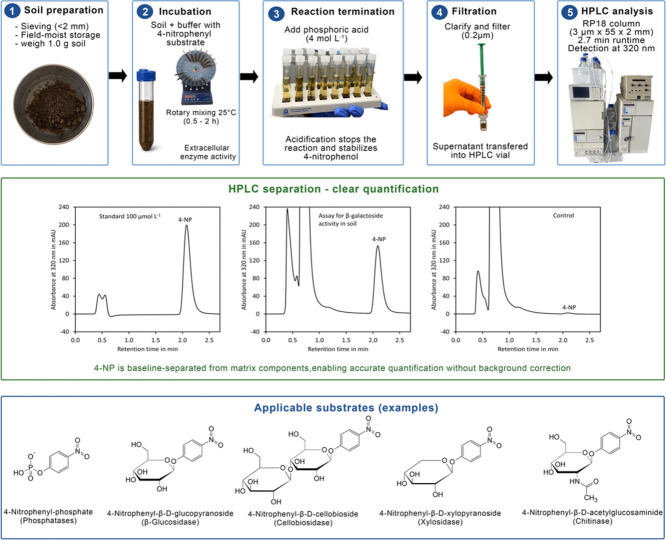

Specifications table

**Table:** 

**Subject area**	Agricultural and Biological Sciences
**More specific subject area**	*Soil Biology and Biochemistry*
**Name of your method**	Using high-performance liquid chromatography in the detection of nitrophenol to quantify soil enzyme activities
**Name and reference of original method**	NA
**Resource availability**	Reagents and equipment are listed in the Materials section

## Background

**Fig. 1:**
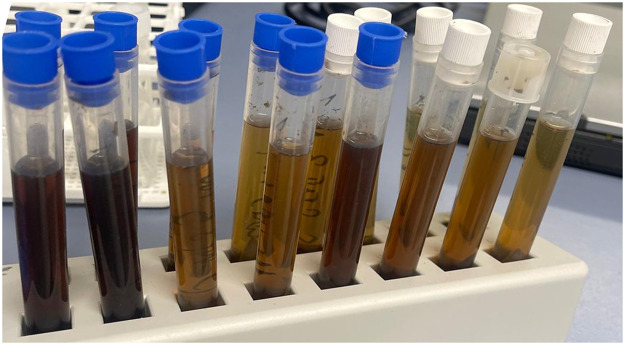
Extracts of humus-rich soils in 100 mmol L^-1^ sodium acetate buffer after addition of calcium chloride solution and alkalinisation with sodium hydroxide.

The activities of marker enzymes provide information on the ability of soils to perform biogeochemical reactions since they are crucial for mineral cycling. In addition, they can be used as indicators for detecting the impacts of anthropogenic actions in agricultural landscapes [1]. The most frequently assessed enzymes are hydrolases including acidic and alkaline phosphatase [2,3], β-glucosidase[4, 5], β-galactosidase [4,5], cellobiosidase [[6], [7], [8]], xylosidase [6], chitinase [6,9], esterase [7,10], protease [6,7,9] and sulfatase [11,12] as well as the oxidoreductases dehydrogenase [[13], [14], [15], [16]], catalase [17], peroxidase [18,19] and phenol oxidase [20,21]. The activity of hydrolases can be measured by incubating soil with a solution of the substrate in a suitable buffer. However, using several natural substrates, for instance cellulose, hemicellulose, and chitin, is hampered by their poor solubility in water and by difficulties in detecting the released products. Thus, many artificial substrates that yield coloured or fluorescent compounds upon hydrolysis have been developed. For detection by spectrophotometry, 4-nitrophenyl and, to some extent, also 2-nitrophenyl derivatives are used. Such artificial substrates yield 4-nitrophenol (4-NP) or 2-nitrophenol upon cleavage, which exhibit an intense yellow colour at a high pH (>10) and can be quantified by measuring the absorbance at approximately 410 nm [22]. However, quantification by spectrophotometry can be affected by interference from organic matter that dissolves in the enzyme assay buffer and usually displays a yellow to brownish colour. Often, calcium chloride is added prior to the addition of sodium hydroxide as a flocculant to precipitate humic acids [22]. Nevertheless, for correction, substrate-free controls must be prepared that allow estimation of the absorbance caused by the organic soil matter. This value is subsequently subtracted from the results measured in the enzyme activity assay [23]. However, this approach is time-consuming and labour-intensive and renders the results less accurate. In humus-rich soils, for instance, chernozem and histosol, the colouring caused by the organic matter may be so intense that spectrophotometric measurement of the liberated 4-nitrophenol is completely impeded (Fig. 1). In principle, fluorogenic substrates, particularly 4-methylumbeliferon-linked artificial substrates [24], can be used to circumvent that problem. However, humic compounds also absorb in the UV range at 340 nm, the wavelength used for excitation of 4-methylumbeliferon (4-MU). For instance, the presence of humic compounds, causing an absorption at 340 nm of only 0.3, reduces the intensity of the excitation light by 50% and thereby causes an underestimation of liberated 4-MU by a factor of 2. Thus, calibration curves must always be prepared in soil extract [1], which is cumbersome and time-consuming. In addition, 4-MU substrates are costly, and thus they are usually used in the microtiter plate format, which is associated with further complications as discussed previously [1,25].

An alternative approach would be the selective detection of 4-NP by its separation from interfering compounds. High-performance liquid chromatography (HPLC) has frequently been used for the analysis of compounds in complex matrices, including plants and soil [[26], [27], [28], [29], [30]] and it has long been used in enzymology, mainly for determining enzyme activities crucial to biochemical and pharmaceutical research [31,32]. In contrast, HPLC has been rarely used for the assessment of soil enzyme activities. Gerritse and Van Dijk [33] were among the first who utilised HPLC for the detection of 4-NP liberated from 4-NP-phosphate by soil enzymes using a column packed with cellulose for separation. Kang and Freeman [34] measured phosphatase activities in soils rich in organic matter by HPLC using methylumbelliferyl-phosphate as a substrate. While these attempts were successful, a clear drawback of HPLC is the significantly increased time required for the analysis of a single sample compared to spectrophotometry, typically exceeding 10 min. However, the development of HPLC particles with small and uniform diameters has dramatically increased separation efficiency [35,36], enabling excellent separation with minimal time and solvent consumption by use of short columns with narrow diameters. Here, we introduce a method for measuring soil enzyme activities using 4-NP-linked substrates by HPLC. The method allows for the separation of the liberated 4-NP from the substrates as well as soil organic matter in 2.7 min. The same HPLC protocol can be used for the measurement of the activities of all 4-nitrophenol-based enzymes, including acidic and alkaline phosphatase, β-glucosidase, cellobiosidase, xylosidase and chitinase. Because 4-NP is baseline-separated, a single calibration curve can be used for all these assays, and substrate-free controls are unnecessary. The method is highly reproducible and shows a linear response over a wide range. Importantly, humic acids are efficiently separated and thus the sensitivity of the method is not affected by the soil matrix. Thus, for humus-rich soils, the method has a much higher sensitivity than the spectrophotometric assay. This allows application of significantly lower incubation times, typically in the range of 0.5 to 2 h, than for the spectrophotometric assay, where an incubation time up to 24 h is frequently used. For the spectrophotometric assay microbiocidal compounds such as chloroform or toluene must be added to prevent bacterial growth during incubation. In the method described here, this is not required due to the short incubation time.

## Method details

### Materials

• Tubes, 10 mL, with caps.
• Syringes, 1 mL.
• Syringe filters, 0.2 µm nylon membrane, 13 mm diameter.
• HPLC vials, 1.5 mL with caps.

### Chemicals

• Acetic acid, glacial, 98–100% (w/w), CAS 64-19-7.
• Acetonitrile, HPLC grade, CAS 75-05-8.
• Citric acid, anhydrous, CAS 77-92-9 or monohydrate, CAS 5949-29-1.
• Maleic acid, CAS 110-16-7.
• Boric acid, CAS 10,043-35-3.
• Hydrochloric acid 37% (w/w), CAS 7647-01-0.
• Phosphoric acid 85% (w/w), CAS 7664-38-2.
• Sodium hydroxide, CAS 1310-73-2.
• Tris(hydroxymethyl)aminomethane (TRIS), CAS 77-86-1.
• 4-Nitrophenol >99%, CAS 100-02-7.
• 4-Nitrophenyl phosphate disodium salt hexahydrate, CAS 4264-83-9.
• 4-Nitrophenyl-β-d-glucopyranoside, CAS 2492-87-7.
• 4-Nitrophenyl-β-d-cellobioside, CAS 3482-57-3.
• 4-Nitrophenyl-β-d-xylopyranoside, CAS 2001-96-9.
• 4-Nitrophenyl-*N*-acetyl-β-d-glucosaminide, CAS 3459-18-5.

### Reagents and solutions

• Phosphoric acid 4 mol L^-1^ (transfer approximately 500 mL distilled water into a 1 L volumetric flask and add slowly 270 mL phosphoric acid 85% (w/w). Shake gently and wait until the solution has cooled to room temperature and add water to a final volume of 1 L).
• Hydrochloric acid 4 mol L^-1^ (transfer approximately 500 mL distilled water into a 1 L volumetric flask and add 333 mL hydrochloric acid 37% (w/w). Wait until the solution has cooled to room temperature and add water to a final volume of 1 L).
• Sodium hydroxide 4 mol L^-1^ (dissolve 160 g sodium hydroxide in approximately 800 mL. Let it cool to room temperature prior addition of water to a final volume of 1 L).
• Buffer No 1: sodium acetate buffer 100 mmol L^-1^ pH 5.0 (transfer 5.55 mL of glacial acetic acid to a beaker containing approximately 900 mL of distilled water. Adjust the pH to 5.0 ± 0.1 using sodium hydroxide 4 mol L^-1^. Transfer the mixture quantitatively into a 1000 mL volumetric flask and add distilled water to the mark. The solution can be kept at room temperature for up to 3 months).
• Buffer No 2: TRIS/HCl buffer 100 mmol L^-1^ pH 9.5 (weigh 12.1 g of TRIS into a beaker and dissolve in approximately 900 mL of distilled water. Adjust the pH to 9.5 ± 0.1 using hydrochloric acid 4 mol l–1. Transfer the solution quantitatively into a 1000 mL volumetric flask and add distilled water to the mark. The solution can be kept at room temperature for up to 3 months).
• Buffer No 3: sodium citrate phosphate buffer 100 mmol L^-1^ pH 6.0 (weigh 19.2 g anhydrous citric acid or 21 g citric acid monohydrate and dissolve in approximately 900 mL distilled water. Add 6.74 mL of phosphoric acid (85%w/w) and adjust the pH to 6.0 using sodium hydroxide 4 mol L^-1^. Transfer the solution quantitatively into a 1000 mL volumetric flask and add distilled water to the mark. The solution can be kept at room temperature for up to 3 months).
• MUB stock solution (modified universal buffer; dissolve 12.1 g TRIS, 11.6 g maleic acid, 14.0 g citric acid and 6.3 g boric acid with water to a total volume of 1 L). Note: boric acid has been classified as a substance of very high concern due to its possible reproductive toxicity. Thus, this solution and its dilutions should be handled with care.
• MUB buffer set to a specific pH (transfer 200 mL MUB stock solution into a 1 L beaker and add water to a volume of approximately 800 mL. Add sodium hydroxide 4 mol L^-1^ or hydrochloric acid 4 mol L^-1^ until the desired pH is reached).
• 4-Nitrophenol stock solution 1000 µmol L^-1^ (transfer 278.2 ± 0.5 mg 4-nitrophenol quantitatively into a 2000 mL volumetric flask, dissolve in approximately 1 L distilled water and add 25 mL phosphoric acid 4 mol L^-1^. Add water to the mark. This solution can be kept at room temperature in the dark for at least 2 years).
• 4-Nitrophenol standard 100 µmol L^–1^ (transfer 10 mL of 4-Nitrophenol stock solution 1000 µmol L^-1^ into a 100 mL volumetric flask, add 2.5 mL phosphoric acid 4 mol L^-1^, and add water to the mark. This solution can be kept at room temperature in the dark for at least 2 years).
• HPLC eluent 20 mmol L^-1^ acetic acid in acetonitrile/water = 30/70 (v/v) (weigh 698.7 g (700 mL) of water into a 1 L glass bottle, add 236.0 g (300 mL) of acetonitrile as well as 1.11 mL of glacial acetic acid).

### Instrumentation/apparatus

• Rotary mixer.
• Analytical balance.
• HPLC system.

### HPLC set up

**Fig. 2:**
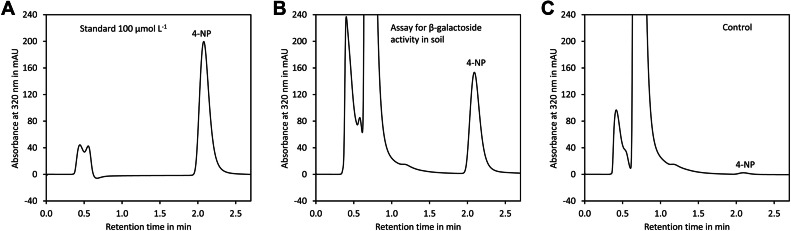
Representative chromatograms. (A) 4-NP standard 100 µmol L^-1^. (B) Chromatogram for a typical assay for β-glucosidase activity in soil. (C) chromatogram of a blank reaction for the assay containing the buffer and substrate but no soil. The small 4-NP peak in this chromatogram originate from an impurity in the substrate.

An LC–10 system (Shimadzu, Kyoto, Japan) equipped with a SCL–10A system controller, an LC–10AD pump, an FCV–10AL eluent valve, a SIL–10A autosampler, a CTO–10ASvp column oven, and *An spd*–10A UV detector was used for HPLC analyses. Chromatographic separations were performed on a Purospher STAR RP–18 endcapped 3 µm particle size HPLC column with 55 mm length and 2 mm inner diameter (Merck Millipore, Billerica, MA, USA). An injection volume of 10 µL was used for all samples and standards. The eluent consisted of 20 mM acetic acid in acetonitrile/water = 30/70 (v/v) and was pumped isocratically at a flow rate of 0.4 mL min^-1^. The 4-nitrophenol peak had its apex at approximately 2 min, and the total analysis time was 2.7 min (Fig. 2). Detection was performed spectrophotometrically at 320 nm. Chromatograms were evaluated with the Clarity software package (DataApex, Prague, Czech Republic).

### Protocol

Notes: 1) The assay must be performed at a constant temperature. Different temperatures are reported in the literature, most frequently 37 °C, but also 30 °C, 25 °C and 20 °C are frequently used. We opted for 20 °C as it is closer to the ambient temperature of typical soils. To maintain a constant temperature, we stored all materials and reagents in a room maintained at 20 °C and conducted the assays there. Alternatively, a water bath can be used, and all materials and reagents should then be kept in the water bath or an oven set to 20 °C.

2) Soil samples should be processed as quickly as possible to avoid losses of enzyme activity. Ideally, soil samples are aliquoted on the day of sampling and stored at −80 °C. However, storage at 4 °C overnight usually has no detectable effect. For the same reason, the samples must be processed field-wet and the dry weight, which is required for the calculation of enzyme activities, must be determined on a second aliquot.

### Sample preparation and determination of the dry weight content

1. Sieve the field-moist soil through a 2 mm sieve.
2. For each enzyme to be measured, weigh 1.00±0.01 g of soil into a 10 mL screw-cap tube and either use immediately or store at −80 °C until analysis.
3. For determination of the dry content, transfer approximately 20 g of the sieved soil into a weighed, empty beaker (mass B).
4. Note the total weight of the beaker plus moist soil (mass BM) to the nearest 0.001 g.
5. Place the beaker into an oven set to 65 °C and dry until constant weight.
6. Determine the weight of the beaker plus the dried soil (mass BD).
7. Calculate the dry weight in percent (DW [%]) using the formula.
  $$DW\;\left[\%\right]=\;\frac{BD-B}{BM-B}\times \;100$$

### Enzyme assay

1. Prepare the substrate solution by dissolving the required amount of substrate in 100 mL buffer (see Table 1).

**Table 1:** Description of soil enzymes with corresponding substrates and assay conditions.

Enzyme	Recom. Buffer^a^	Substrate	Recom. concentration	Weight of substrate for 100 mL buffer	Default incubation time
Acid phosphatase	No. 1	4-Nitrophenyl phosphate disodium salt hexahydrate	2–5 mmol L^–1^	74.2 ± 1.0 mg (2 µmol l^–1^) 185.6 ± 2.0 mg (5 µmol L^–1^)	0.5 h
Alkaline phosphatase	No. 2	4-Nitrophenyl phosphate disodium salt hexahydrate	2–5 mmol L^–1^	74.2 ± 1.0 mg (2 µmol l^–1^) 185.6 ± 1.0 mg (5 µmol L^–1^)	1 h
β-Glucosidase	No. 1	4-Nitrophenyl-β-d-glucopyranoside monohydrate	1 mmol L^–1^	30.1 ± 1.0 mg	2 h
Cellobiosidase	No. 3	4-Nitrophenyl-β-d-cellobioside	1 mmol L^–1^	46.3 ± 1.0 mg	2 h
β-Xylosidase	No. 3	4-Nitrophenyl-β-d-xylopyranoside	2–10 mmol L^–1^	54.2 ± 1.0 mg (2 µmol L^–1^) 271.2 ± 2.0 mg (10 µmol L^–1^)	2 h
Chitinase	No. 3	4-Nitrophenyl-*N*-acetyl-β-d-glucosaminide	1 mmol L^–1^	34.2 ± 1.0 mg	2 h

a Recom., recommended. If a buffer system with a pH identical to that of the soil sample is preferred, MUB set to the desired pH using NaOH may be used. In principle, other buffer systems may be used if they are suitable for the enzymatic reaction.
2. Pre-warm the substrate solution to 20 °C for at least 1 hour. Note: as mentioned above, temperature has a major impact on enzyme activity; therefore, all materials must be equilibrated to the assay temperature.
3. Add 5 mL of the substrate solution to each tube containing 1 g of soil, and then close the tubes with caps. Prepare also one blank tube (using only 5 mL substrate solution without soil). Note: The sample may be fumigated with chloroform or toluene to kill microorganisms by adding 100 µL of this solvent to the tube containing the soil sample. However, due to the low background, the HPLC method presented here has a significantly increased sensitivity for 4-NP, allowing for much shorter incubation times (0.5 to 2 h) than in the classical assays (up to 24 h). Consequently, mainly extracellular enzymes are measured, and the results obtained with or without chloroform or toluene are essentially identical, making fumigation dispensable.
4. Incubate on a rotary shaker at 30 rpm at 20 °C for the required amount of time (see Table 1 for default values). Note: the time may be adapted according to the expected enzymatic activity.
5. Stop the reaction by adding 0.5 mL of phosphoric acid (4 mol L^-1^) and mix well.
6. Allow the soil particles to settle for 5–10 min before filtering approximately 1 mL supernatant through a 0.2 µm nylon syringe filter directly into an HPLC vial. Optionally, the soil samples can be centrifuged before filtration, although this is usually not necessary.
7. Determine the concentration of 4-nitrophenol using an HPLC system equipped with a LiChroCART RP18, 3 µm, 55 × 2 mm separation column using isocratic elution with 20 mmol L^-1^ acetic acid in acetonitrile (ACN)/water = 30/70 (v/v) as outlined below.
8. Establish a calibration curve by injecting 20 µL, 15 µL, 10 µL, 5 µL, 2 µL, and 1 µL of a 4-nitrophenol standard at 100 µmol L^-1^. If 10 µL samples are injected, these injection volumes correspond to nitrophenol concentrations of 200, 150, 100, 50, 20, and 10 µmol L^-1^, respectively. Note: alternatively, solutions for the concentrations stated above can be injected at a fixed volume of 10 µL.
9. Next, inject the blank and the samples. The blank should be measured at the beginning, after every 20 samples and at the end of the series. Note: repeated measurements of the blank are essential when using the substrate 4-nitrophenyl phosphate (see note to step 18). In contrast, a single blank measurement is sufficient for the other substrates listed in Table 1.
10. Establish a standard curve and calculate the amount of 4-nitrophenol released.
11. Peak areas from the negative controls are subtracted from those of the soil assays. Note: 4-nitrophenlyphosphate hydrolyses in the presence of phosphoric acid relatively quickly. Thus, the blank value must be estimated for every sample using the blank samples. This can be done by the Excel spreadsheet present in the Supplementary Material. The other substrates listed in Table 1 are stable in the presence of 100 mol L^-1^ phosphoric acid for at least 10 h. Nevertheless, blanks are still required since commercially available substrates may contain small quantities of free 4-NP.
12. Express the enzyme activity as µmol of 4-nitrophenol released per gram of soil per hour.

### Calculations and statistical analyses

**Fig. 3:**
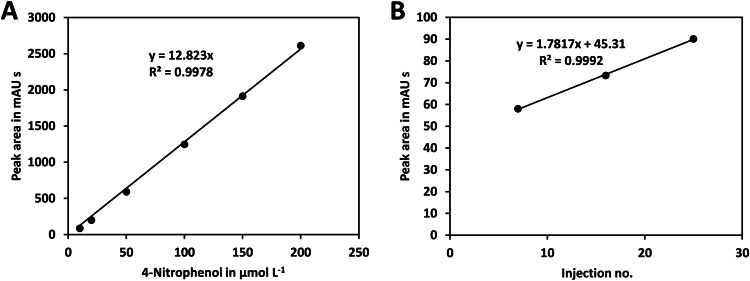
Evaluation of HPLC data. (A) Calibration curve for authentic 4-NP. (B) An injection number-specific calibration curve for the calculation of the blank for each sample. The interception originates mainly from 4-NP present in the substrate as impurity, while the slope is caused by H^+^-catalysed substrate cleavage.

Calibration curves obtained for peak areas vs. 4-NP concentration typically have a very small interception close to zero. Since the sample peak areas must be corrected for the blank, we decided to simplify the calculation of the 4-NP concentrations by just using the slope. Thus, for calculation of the 4-NP calibration curve, the interception was set to zero (Fig. 3A).

All 4-nitrophenyl substrates contain small amounts of 4-NP. In addition, some 4-NP substrates are slightly acid labile and thus small amounts of 4-NP are liberated while the samples are waiting to be injected. Thus, blank samples must be analysed at defined intervals, ideally before the first, after approximately every 20 and after the last sample. Their readings were used to calculate an injection number-specific correction curve (Fig. 3B). The slope and intercept of this regression line were used to predict a blank value for each sample. This value was then subtracted from the observed sample peak area to get the corrected peak. The soil enzyme activity was finally calculated from the 4-NP concentration by using the following formula:

$$Enzyme\;activity\left(\mu \text{mol}g^{-1}h^{-1}\right)=\frac{4-\text{NP}\;\left(\mu \text{mol}\;L^{-1}\right)\;x\;\text{total}\;\text{reaction}\;\text{volume}\left(\text{mL}\right)\;x\;0.1}{\text{incubation}\;\text{time}\;\left(h\right)\;x\;\text{soil}\;\text{wet}\;\text{weight}\;\left(g\right)\;x\;\text{soil}\;\text{dry}\;\text{content}\;\left(\%\right)\;}$$

**Fig. 4:**
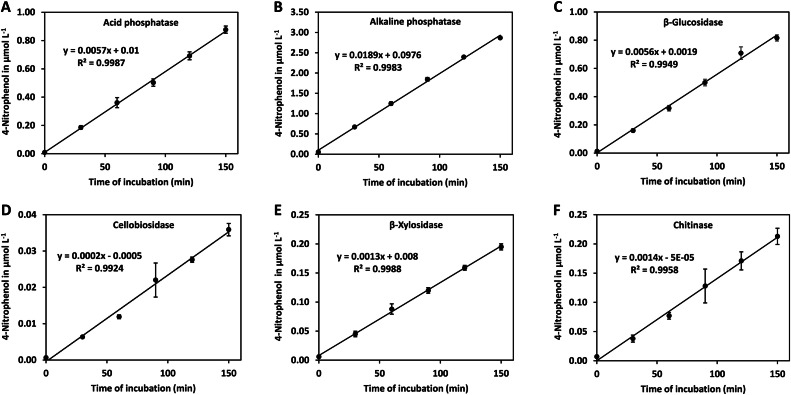
Time-course analysis showing the linear formation of 4-NP with increasing incubation time for (A) acid phosphatase, (B) alkaline phosphatase, (C) β-glucosidase, (D) cellobiosidase, (E) β-xylosidase, and (F) chitinase. Each substrate was used in a concentration of 2 mmol L^–1^. Each point on the graphs represents the mean, and the bars the standard deviation of four replicates. Soil samples were taken from an agricultural field in Saxony-Anhalt at a depth of 0 to 30 cm using a soil corer of 2 cm diameter. The samples were stored at 4 °C for 24 h temporarily, then sieved through a 2 mm sieve and aliquots of 1.00 g were stored at −80 °C until enzyme activity measurement.

**Fig. 5:**
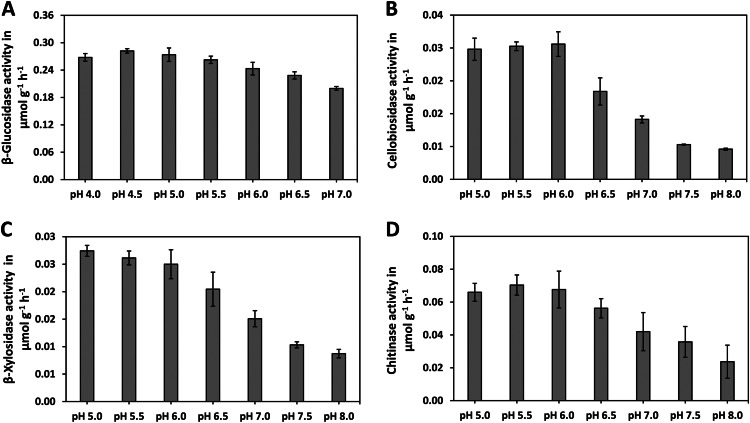
Effect of pH on the activity of four soil enzymes using HPLC-based 4-NP detection method. (A) β-Glucosidase, (B) cellobiosidase, (C) β-xylosidase, and (D) chitinase. Soil enzyme activities were determined from pH 4.0 to pH 8.0 using a buffer consisting of 100 mmol L^–1^ citric acid in 100 mmol L^–1^ phosphoric acid set with sodium hydroxide to the indicated pH. For all substrates, a final concentration of 2 mmol L^–1^ was used. Columns represent the average, and error bars represent the standard deviations of three replicates.

**Fig. 6:**
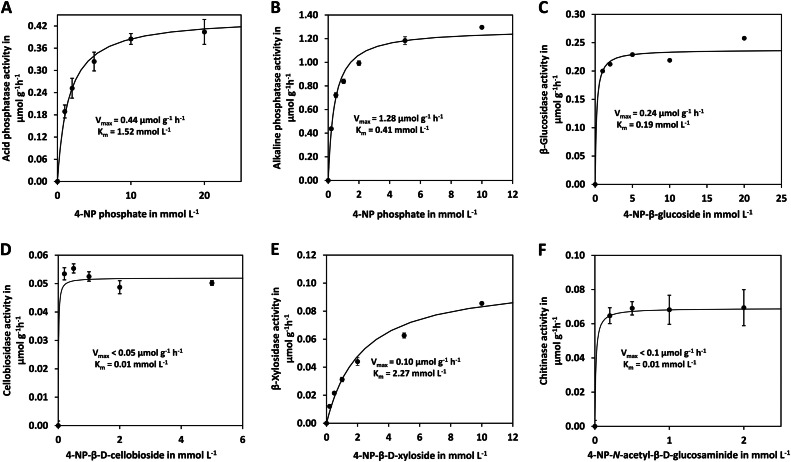
Michaelis-Menten graphs showing the relationship between substrate concentration and enzyme activity for representative soil enzymes. (A) Acid phosphatase, (B) alkaline phosphatase, (C) β-xylosidase, (D) cellobiosidase, (E) chitinase, and (F) β-glucosidase. Points represent the average, and error bars the standard deviation of four replicates.

Statistical analysis was conducted using Microsoft Excel 2021 (Microsoft Corporation, 2021). A spreadsheet for analysis is available in the Supplementary Material section. To test precision, we ran four technical replicates of the same soil sample. Variation among replicates was quite low, as indicated by the small standard deviations (error bars) obtained for most samples shown in Fig. 4, Fig. 5, Fig. 6.

## Method validation

### Linearity test of product (4-NP) formation over time

The first step in validating the method was to confirm that 4-NP production increased linearly over time under the specified assay conditions. Soil samples were incubated with the appropriate substrates and buffer solutions. The reactions were stopped at defined intervals: 0 min (immediate termination), 30 min, 60 min, 90 min, 120 min, and 150 min (Fig. 4). These time points were selected from preliminary kinetic tests for each target enzyme. As illustrated in Fig. 4, 4-NP levels increased steadily over time, indicating that the reaction progressed consistently throughout the incubation period.

### Dependency of the enzyme activity on pH

Soil enzymatic activity is strongly influenced by pH, which varies significantly depending on soil type and land management practices. To demonstrate the effect of soil pH on enzyme activities, we used the buffer system 3 adjusted to different pH values. Each enzyme assay was performed in triplicate across the pH range under controlled laboratory conditions. The pH value that yielded the highest product formation was selected as the optimal condition for subsequent soil enzyme assays. For example, as shown in Fig. 5, β-glucosidase, cellobiosidase, β-xylosidase, and chitinase exhibited the highest activities close to pH 5 with broad pH optima, which aligns with previous reports [6,[37], [38], [39], [40]]. We did not perform this test for phosphatases since acid and alkaline phosphatase are assayed in buffers of different pH values. However, the pH optimum of the soil enzymes may depend on the soil pH. To account for that, it is possible to use modified universal buffer (MUB) set to the soil pH instead of using the buffers stated above.

### Impact of the substrate concentration on the reaction rate

To further refine the assay conditions, we evaluated how substrate concentrations influenced enzyme kinetics using the Michaelis-Menten model. Depending on the enzyme, we tested substrate concentrations in the range of up to 20 mmol L^–1^. The Michaelis-Menten constant (K_m_) and maximum velocity (V_max_) were calculated by regression analysis using the Michaelis-Menten equation [41]. The enzymatic activities expressed in µmol 4-NP released per g soil (dry weight) and h were plotted against the substrate concentrations to produce the kinetic curves (Fig. 6). As substrate levels increased, enzyme activity rose and then levelled off, indicating saturation. All enzymes followed Michaelis-Menten kinetics but quite distinct K_m_ values ranging from <0.05 mmol L^–1^ for cellobiosidase to 2.3 mmol L^–1^ for β-xylosidase were obtained. For the latter substrate, complete saturation was not reached even at the highest substrate concentration tested.

Ideally, the substrate concentration used for the assay should be close to substrate saturation. However, most substrates have limited solubility in water, and they are costly. In addition, some substrates, particularly 4-NP phosphate, are sensitive to H^+^-catalysed cleavage, which causes increased blanks when high concentrations are used, limiting the sensitivity of the assay. Thus, it is recommended to work at concentrations as low as possible. For β-glucosidase, cellobiosidase and chitinase, 4-NP-substrate concentrations of 1 mmol L^–1^ are sufficient. For the phosphatases, 5 mmol l^–1^ would be ideal. However, if the results are just used for comparison, 2 mmol L^–1^ is sufficient, which has the advantage of a lower background. For β-xylosidase, a substrate concentration 10 mmol L^–1^ would be ideal. However, this substrate is particularly costly. Thus, 2 mmol L^–1^ may be used if the results are just used for comparison, for instance, between two sites. However, under such conditions, only a small portion of the substrate (<10%) should be cleaved to avoid inaccuracies due to reduced enzyme activity caused by lower substrate concentrations. In any case, the utilised substrate concentrations should be reported along with the results.

### Comparison of the spectrophotometric and HPLC methods regarding sensitivity and specificity

The assay for β-glucosidase activity was employed to determine the limits of detection (LOD) and quantification (LOQ) for the liberated 4-NP. The LOD and LOQ were defined as the 3- and 10-fold standard deviation (SD) of the blank, respectively, and were determined according to section 3.2.3.2 of the ICH guideline Q2(R2) on Validation of Analytical Procedures. For each type of analysis 20 independent samples were measured. In the absence of interfering compounds, HPLC showed slightly enhanced sensitivity compared to spectrophotometry (0.15 µmol L^–1^ vs. 1.1 µmol L^–1^; see Table 2). To estimate the LOD and LOQ in the presence of matrix interferences, blank extractions (buffer without substrate) were prepared from field soil samples with low (1.2% (w/w)), medium (3.7% (w/w)), and relatively high humus contents (9.5% (w/w)) and subsequently analysed by both methods. The resulting average background signals, their standard deviations, and the calculated LODs and LOQs are summariszed in Table 2. The high absorbance values of the background controls demonstrate that the spectrophotometric assay lacks specificity for 4-NP. Furthermore, its LOD and LOQ increased dramatically with the humus content of the soil. This trend is driven by the high standard deviations of the blank reactions, which likely originate from micro-inhomogeneities within the soil samples. This severely impairs the sensitivity of the spectrophotometric method, necessitating prolonged incubation times to generate detectable amounts of the coloured 4-NP product. In sharp contrast, the HPLC-based method remained largely unaffected by the humus content, as evidenced by the consistent background peak areas (Table 2). Similarly, the LOD and LOQ of the HPLC method were independent of the humus content, maintaining high sensitivity with an LOQ of approximately 0.1 µmol L^–1^ across all tested soils. These data confirm that no blank control is required for the method presented here. In addition, the high specificity maintains assay sensitivity even in matrix-rich samples. Consequently, even minor increases in the 4-NP concentration can be reliably quantified, allowing for short incubation times (0.5–2 h). In addition to the increased speed this has the advantage the advantage that accurate results are obtained even in the absence of microbiocidal agents that must be added to reactions with prolonged incubation times. In summary, the high specificity of the HPLC-based method maintains high sensitivity for humus-rich samples, which allows for the application of short incubation times and increases the accuracy of the results.

**Table 2:** LOD and LOQ for 4-NP of the spectrophotometric and HPLC-based assay in absence and presence of soil.

Sample type	Spectrophotometry				HPLC	
	ABS±SD AU	LOD µmol L^–1^	LOQ µmol L^–1^	Area±SD mAU s	LOD µmol L^–1^	LOQ µmol L^–1^
Blank (no soil)^a^	0.033±0.002	0.3	1.1	2.48±0.20	0.05	0.15
Soil, 1.5% (w/w) humus^b^	0.059±0.013	2.3	7.6	0.56±0.16	0.04	0.12
Soil, 3.7% (w/w) humus^b^	0.103±0.040	7.2	23.9	0.43±0.13	0.03	0.10
Soil, 9.5% (w/w) humus^b^	0.613±0.063	11.3	37.5	0.50±0.18	0.04	0.14

a Assay buffer containing 1 mmol L^–1^ 4-NP-β-d-glucoside was used.

b Assay buffer without substrate was used for extraction. The humus content was calculated from the content of organic carbon multiplied by 1.724. The content of organic carbon was determined as described previously [42].

## Limitations

A limitation of this assay is the H^+^-catalysed cleavage of the substrates, which is an issue particularly for 4-NP phosphate. For the spectrophotometric assay, this is not a problem because all samples and standards are measured at approximately the same time. In contrast, analysis of a single sample by HPLC takes 2.7 min, thus analysing a whole set of samples may take a few hours. Therefore, blanks must be measured regularly during sample analysis, and a blank value should be calculated for every run. However, for the other substrates listed in Table 1, this is much less of an issue because they are quite stable under acidic conditions. If the reaction products need to be analysed the next day, it is recommended to keep them in the freezer (−20 °C) to minimise acid-catalysed 4-NP liberation. It is important to note that the reaction temperature must be carefully controlled since a few degrees Celsius deviation can have a huge impact on the enzymatic activity.

## Ethics statements

Not applicable.

## Declaration of generative AI and AI-assisted technologies in the writing process

During the preparation of this work, the authors utilised Grammarly and ChatGPT to enhance clarity and readability. After using this tool, the authors reviewed and edited the content as needed and take full responsibility for the content of the publication.

## Author contributions

WI: conceptualization, methodology, investigation, data curation, validation, visualization, writing – original draft preparation, writing – review and editing. JG: supervision, funding, writing – review and editing. AK: supervision, funding, writing – review and editing. WR: conceptualization, methodology, investigation, validation, visualization, funding, writing – original draft preparation, supervision, writing – review and editing. All authors have read and agreed to the published version of the manuscript.

## Funding

This study was funded by the 10.13039/501100001659Deutsche Forschungsgemeinschaft (DFG, German Research Foundation), project number 528485254 – FIP 16 (AgriRestore). We acknowledge the support of the DEAL consortium for covering the costs of Open Access publication.

## Declaration of competing interest

The authors declare that they have no known competing financial interests or personal relationships that could have appeared to influence the work reported in this paper.

## Data Availability

Data will be made available on request.

## Appendix Supplementary materials

Image, Spreadsheet for calculation of enzyme activities.
